# Reduced orbitofrontal cortical thickness in male adolescents with internet addiction

**DOI:** 10.1186/1744-9081-9-11

**Published:** 2013-03-12

**Authors:** Soon-Beom Hong, Jae-Won Kim, Eun-Jung Choi, Ho-Hyun Kim, Jeong-Eun Suh, Chang-Dai Kim, Paul Klauser, Sarah Whittle, Murat Yűcel, Christos Pantelis, Soon-Hyung Yi

**Affiliations:** 1 Melbourne Neuropsychiatry Center, Department of Psychiatry, University of Melbourne and Melbourne Health, Parkville, Victoria, Australia; 2 Florey Institute of Neuroscience and Mental Health, Parkville, Victoria, Australia; 3 Division of Child and Adolescent Psychiatry, Department of Psychiatry, College of Medicine, Seoul National University, Seoul, Republic of Korea; 4 Department of Child Development and Family Studies, College of Human Ecology, Seoul National University, Seoul, Republic of Korea; 5 Interdisciplinary Program (Early Childhood Education Major), College of Education, Seoul National University, Seoul, Republic of Korea; 6 Center for Campus Life & Culture, Seoul National University, Seoul, Republic of Korea; 7 Department of Education (Educational Counseling Major), College of Education, Seoul National University, Seoul, Republic of Korea

**Keywords:** Internet addiction, Magnetic resonance imaging, Cortical thickness, Orbitofrontal cortex

## Abstract

**Background:**

The orbitofrontal cortex (OFC) has consistently been implicated in the pathology of both drug and behavioral addictions. However, no study to date has examined OFC thickness in internet addiction. In the current study, we investigated the existence of differences in cortical thickness of the OFC in adolescents with internet addiction. On the basis of recently proposed theoretical models of addiction, we predicted a reduction of thickness in the OFC of internet addicted individuals.

**Findings:**

Participants were 15 male adolescents diagnosed as having internet addiction and 15 male healthy comparison subjects. Brain magnetic resonance images were acquired on a 3T MRI and group differences in cortical thickness were analyzed using FreeSurfer. Our results confirmed that male adolescents with internet addiction have significantly decreased cortical thickness in the right lateral OFC (*p*<0.05).

**Conclusion:**

This finding supports the view that the OFC alterations in adolescents with internet addiction reflect a shared neurobiological marker of addiction-related disorders in general.

## Introduction

Internet addiction has been increasingly recognized as a mental disorder. Recent estimates of its high prevalence in young people, combined with evidence that problematic internet use is a maladaptive behavior with potentially serious occupational and mental health consequences, support the validity of the diagnosis [1]. Nevertheless, there has been much disagreement in the planning for DSM-V about how to conceptualize this relatively new condition, or its core psychopathology [2]. Given this background, identifying the existence of any biological markers would help improve the diagnostic validity [3].

The neural basis of substance addiction has been more extensively studied and is better established compared to other forms of ‘addiction’ (e.g., behavioral addictions). So far, numerous studies in the literature have implicated the role of the orbitofrontal cortex (OFC) in addiction [4-6]. Volkow and colleagues (2000, 2002) suggest that the OFC is one of the most implicated frontal cortical areas in drug addiction [4,7]. Recently, researchers in our group have reported a longitudinal prospective study result suggesting that structural abnormalities in the OFC might predate and contribute to risk for later cannabis use in young people [8]. Similarly, although few studies have been conducted, the reported functional and structural alterations observed in internet addiction have been relatively consistent in demonstrating altered OFC function (especially in the right hemisphere) and structure [9-14].

We hypothesized that adolescents with internet addiction would show structural abnormalities of the OFC, preferentially in the right hemisphere. Specifically, we conducted a case–control comparison of cortical thickness in adolescents with and without internet addiction, particularly focusing on excessive online gaming, which is among the major subtypes of this disorder [15].

## Materials and methods

### Subjects

Fifteen right-handed male adolescents with internet addiction were recruited through advertisement in Seoul National University Hospital. The recruitment was performed between February and June 2011. In order to establish the diagnosis of internet addiction we used the Young Internet Addiction Scale (YIAS) [16]. In addition, participants were confined to those reporting to have experienced typical components of addiction with their online gaming including: tolerance, withdrawal, preoccupation with playing it, repeated unsuccessful attempts to reduce or stop it, negatively influenced mood when attempting to reduce it, and neglecting important relationships or activities because of it [17,18]. In order to exclude any comorbid psychiatric disorders, we used the Kiddie-Schedule for Affective Disorders and Schizophrenia-Present and Lifetime Version (K-SADS-PL) [19]. Healthy male adolescents were recruited through advertisement in local schools and were screened using the same assessment tools described above. Exclusion criteria for both groups were any axis I psychiatric disorder including substance abuse, epilepsy or other neurological disorders, and past history of severe head trauma. The prevalence of internet addiction has been estimated to be much higher in males than in females [1]. Given that males are over-represented in internet-addicted populations, and given time and budget limitations, we decided to focus only on male adolescents. This study was approved by the institutional review board for human subjects at the Seoul National University. All the adolescents and their parents provided written informed consent prior to study entry.

### Data acquisition

Whole brain T1-weighted MR images were acquired on a 3T Siemens scanner (Siemens Magnetom Trio Tim Syngo MR B17, Germany) with the following parameters: TR 1900 ms; TE 2.36 ms; inversion time 700 ms; flip angle 9°; voxel size 1.0 mm^3^; slices 224. Head motions were minimized by filling the empty space around the head with sponge material and fixing the lower jaw with tape.

### Image processing

Cortical thickness was estimated using FreeSurfer 5.1.0 (Massachusetts General Hospital, Boston, MA, US), a set of software tools that provides a semi-automated method to investigate brain morphometry. The surface-based stream involves (i) normalization of brain signal intensity, (ii) skull-stripping, (iii) segmentation of gray and white matter, (iv) delineation of the gray-white interface (inner surface), and (v) tracing of the pial (outer) surface. The distance between equivalent vertices in these two surfaces represents the cortical thickness. The whole cortex of each subject was visually inspected and systematically corrected for errors in a blind manner to the group status of the participants. We used our sample to generate an average target surface and the data for each participant were pre-smoothed with a full-width half-maximum Gaussian kernel of 10 mm prior to statistical analyses.

### Data analysis

Region-of-interest (ROI)-based analyses were conducted comparing the cortical thickness of the OFC generated by FreeSurfer based on the Desikan-Killiany atlas [20]. More specifically, the Desikan-Killiany atlas defines the lateral and medial divisions of the OFC. The rostral/caudal and medial/lateral boundaries of these two structures are the rostral extent of the lateral orbital gyrus/the caudal portion of the lateral orbital gyrus and the midpoint of the olfactory sulcus/the lateral bank of the lateral orbital sulcus (and/or the circular insular sulcus) for the lateral OFC; and the rostral extent of the medial orbital gyrus/the caudal portion of the medial orbital gyrus (or gyrus rectus) and the cingulate cortex/the medial bank of the superior frontal gyrus for the medial OFC, respectively [20]. Analysis of covariance (ANCOVA) using the general linear model (GLM) was performed with SPSS 19.0 (SPSS Inc., Chicago, IL, USA), and the main effect of group (internet addiction vs. healthy control) was analyzed controlling for age, intelligence quotient (IQ), and intracranial volume (ICV). Results of this analysis were reported with a significance threshold of *p*<0.05 (two-tailed).

## Results

### Participant characteristics

The groups of adolescents with and without internet addiction significantly differed in age (13.33±2.84 for internet addiction; 15.40±1.24 for control; *p*=0.018). IQ was comparable across both groups (103.80±15.84 for internet addiction; 109.06±9.84 for control; *p*=0.283), and a significant difference in ICV was found (1434.42±158.33 cm^3^ for internet addiction; 1577.21±183.12 cm^3^ for control; *p*=0.030). The YIAS score was significantly higher in the internet addiction group (57.26±16.11 for internet addiction; 37.60±9.72 for control; *p*=0.000).

### ROI-based analyses

The analysis revealed four ROIs with significant differences in cortical thickness (*p*<0.05), which were the lateral OFC, isthmus of the cingulate cortex, and pars orbitalis in the right hemisphere and lateral occipital cortex in the left hemisphere, all displaying cortical thinning in adolescents with internet addiction compared to healthy controls (Table 1).

**Table 1:** Parcellated region-of-interest-based comparison of cortical thickness between adolescents with internet addiction and healthy controls

**Comparison**	**Region**	**Side**	**Cortical thickness (mm)**		**F**	***p*-value**	**R**^**2**^
			**Internet**	**Control**			
Internet < Control	Lateral orbitofrontal	Right	2.78±0.21	2.80±0.18	6.609	0.016	0.395
Internet < Control	Cingulate (isthmus)	Right	2.81±0.31	2.84±0.26	6.813	0.015	0.437
Internet < Control	Lateral occipital	Left	2.48±0.15	2.48±0.08	9.323	0.005	0.581
Internet < Control	Pars orbitalis	Right	2.75±0.20	2.84±0.32	5.266	0.030	0.258

Cortical thickness is given as mean ± standard deviation.

### Secondary analysis

**Figure 1:**
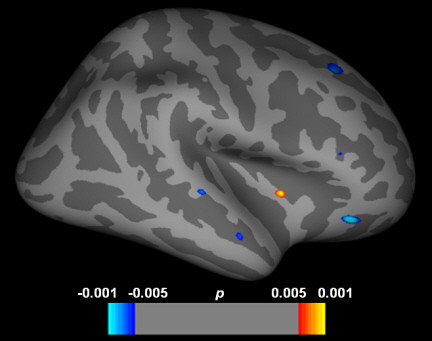
**Vertex-wise whole brain comparison of cortical thickness between adolescents with internet addiction and healthy controls.** Red color indicates cortical thickness is greater in adolescents with internet addiction, and blue color indicates cortical thickness is greater in controls.

To provide a complementary perspective of our finding, a surface-based whole brain analysis using FreeSurfer’s Qdec (version 1.4) was performed by fitting a between-subject GLM at each surface vertex to compare cortical thickness between the groups (uncorrected, *p*<0.001). As illustrated in Figure 1, reduction in lateral OFC thickness was replicated by this analysis.

## Discussion

This is the first structural brain imaging study of cortical thickness in adolescents with internet addiction. Consistent with our hypothesis, the results indicated a reduced thickness of OFC in the internet addiction group compared to healthy controls. This finding is in accord with the results of former neuroimaging studies of internet addiction [9-14], and supports the theoretical model of addiction disorders, which emphasizes the involvement of the OFC.

The current finding on internet addiction supports the results of former studies on substance addiction, including ours [8], that have argued that the right OFC plays an important role in the biological mechanism of addiction disorders more broadly. The result of this study is not only in line with numerous other findings in the literature implicating the role of the OFC in addiction [4-6], but also with those indicating that this brain region in the right hemisphere might be particularly important [21].

In this study, only the lateral and not the medial OFC was shown to be significantly different in adolescents with internet addiction. The reason for this finding is not clear, but there have been multiple studies reporting different functions between the lateral and medial OFC, especially in reward-associated decision-making [22]. For example, the medial OFC has been found to be preferentially activated in choices involving immediate rewards, whereas the lateral OFC has been implicated in choices concerning delayed rewards or suppression of previously rewarded responses [23,24]. It is noteworthy that pars orbitalis, which is laterally adjacent to the lateral OFC, also showed significant cortical thinning in adolescents with internet addiction. This finding supports that the cortical thinning is particularly located in the lateral part of the OFC, without or less involving medial OFC. Further work is warranted on potentially different lateral and medial OFC functions.

Lateral OFC has also been implicated in cognitive flexibility deficits and in the genesis of pathological habits [25]. In this regard, Chamberlain and colleagues (2008) demonstrated that the lateral OFC can be central to neurobiological models of obsessive-compulsive disorder (OCD) [26]. Behavioral addictions are often considered to share similar features with other known disorders including OCD [2], involving specific difficulties in refraining from a certain behavior that causes serious personal consequences. Rotge and colleagues (2008, 2010), based on their previous meta-analyses [27,28], investigated overlapping brain regions between the anatomical and functional brain maps that showed significant gray matter density change and activity during symptom provocation, respectively, in patients with OCD: the authors found that the only overlapping brain region was the lateral OFC. Recently, Zhou and colleagues (2012) have shown impaired mental flexibility along with poor response inhibition in young adults with internet addiction [29]. The implications of reduced cortical thickness in lateral OFC, in relation to its role in internet addiction as well as other conditions with similar neurobehavioral features, are subject to future studies.

The present study has some important limitations. Above all, a different age distribution between the groups was a critical limitation of this study. However, previous reports on normal brain development have shown that cortical thickness peaks at approximately 8–9 years of age and then global cortical thinning begins afterward [30]. Of note is that all the participants in our study were over this age. Therefore, in adolescents, younger individuals tend to have thicker cortex; our finding of thinner right OFC in the younger internet addiction group thus suggests that the age difference in groups was unlikely to have affected the results. Second, we did not measure the duration of internet addiction. Third, the study participants in the internet addiction group were excessive online gamers, and therefore, the current findings may entail limited generalizability to other subtypes of internet addiction [15].

In summary, the results of the present study indicate preliminary findings of reduced thickness of the right OFC in adolescents with internet addiction. The findings are further suggestive of a shared neurobiological mechanism between internet addiction and other addictive disorders.

## Competing interests

All authors declare that they have no competing interests.

## Authors’ contributions

SBH conducted the data analyses and wrote the first draft of the manuscript. JWK, CDK, and SHY were responsible for the study concept and design. SBH, EJC, HHK, and JES were responsible for acquisition of clinical and imaging data. PK and SW assisted with imaging data analyses. PK, SW, MY, and CP contributed to the final version of the manuscript. JWK, CDK, MY, CP, and SHY helped in data interpretation and provided important intellectual content. All authors critically reviewed the content and approved final version submitted for publication.
